# Sources and Level of Nutrition Knowledge Among Adults in Lahore: A Cross-Sectional Descriptive Study

**DOI:** 10.7759/cureus.44186

**Published:** 2023-08-27

**Authors:** Kinza Imran, Qaisar Raza, Hinza Saleem, Rakhshanda Batool

**Affiliations:** 1 Department of Food Science and Human Nutrition, University of Veterinary and Animal Sciences, Lahore, PAK

**Keywords:** level of nutrition knowledge, lahore, adults, basic nutrition knowledge, sources of nutrition knowledge

## Abstract

Objective: This study was aimed at identifying the sources and basic nutrition knowledge among the citizens of Lahore.

Methods: This cross-sectional study was conducted in the metropolis city of Lahore, Pakistan. Four hundred and seventy-six adult citizens of Lahore participated. A random sampling method was used to collect data through a food frequency questionnaire consisting of 23 questions. The questionnaire designed for this study contained questions about demographics, sources of nutrition information, the reliability of these sources of nutrition information, and basic nutrition knowledge. The highest possible score of the questionnaire was marked as 15, and a mean value of 7.5 was used to categorize the low and high values for variables like nutrition knowledge.

Results: The majority of participants (34.2%) took nutrition information from their families, healthcare professionals (23.9%), and online resources (23.5%). The most reliable source for nutrition information was healthcare professionals (78.6%). Many participants (65.30%) had high nutrition-related basic knowledge, and 34.70% had low knowledge. Although more than 50% of participants reported having high nutrition knowledge, but they were not aware of how to read food labels. Those citizens who referred to online resources for information about nutrition knowledge usually got better results regarding nutrition knowledge.

Conclusion: There is a need for more extensive research to identify the quality of sources for nutrition information to form better policies and plans that can be integrated and adopted at the community and national levels to increase the overall nutrition knowledge of people.

## Introduction

Nutrition knowledge is responsible for developing healthy eating habits and improving the nutrition status of people [1]. An enhanced nutrition knowledge and nutrition status improves the health of the population, which ultimately results in increased economic growth for the country. In addition, previous research has shown that improving the health of individuals in a country can improve the gross domestic product of the country [2]. The assessment of nutrition knowledge can help us design and implement health promotion activities [3]. However, finding a single assessment tool for determining nutrition knowledge is not possible in a diversified urban population, so a series of modified questions are needed to assess the basic nutrition knowledge of the population of one city or district. These questions include knowing different terminologies, quantities, and the sources of this information. Having adequate nutrition knowledge is also linked to optimized nutritional behaviors that are important for a healthy lifestyle and disease prevention [4,5]. Therefore, having reliable nutrition information can act as a pivotal point for satisfying nutritional behaviors and habits [6]. For example, a person who knows how to read and interpret the nutrition fact panels on different food products will be more aware and, therefore, more careful in choosing the product as compared to a person who does not have sufficient nutrition knowledge [7,8]. Moreover, the questionnaire designed in this study contained questions about sources of nutrition information that involved certain demographics (ethnicity including Punjabi, Sindhi, Balochi, Pathan, and highest education qualification including Matriculation, Intermediate, and Graduation) and media responsible for the dissemination of information, including online resources (e.g., Google searches, YouTube, etc.) and traditional media (e.g., radio, television, newspapers, etc.) [9-11]. The usage of different media for access to information depends on the different psychological and financial prospects of the community. For instance, people belonging to a lower economic status did not know about fortification (60.29%) and nutrition fact panels (56.9%) due to a lower literacy rate as compared to people who had a better economic status and literacy rate [12]. However, nutrition information for adults belonging to developing countries in Pakistan is limited [13,14]. There is a need to gather data on nutrition information from different areas of Pakistan to develop and implement targeted interventions for health promotion and better nutrition status within communities. This study is aimed at identifying the sources and basic nutrition knowledge among the citizens of Lahore.

## Materials and methods

This was a cross-sectional study conducted in Lahore, Pakistan. Data was collected through the use of a questionnaire from January 2020 to June 2020. Random sampling was used to collect the data. An undergraduate team of five students from the Food Science and Human Nutrition department at the University of Veterinary and Animal Sciences participated in this research. They collected data from different sites in Lahore, including shopping malls, grocery stores, local markets, parks, and cafeterias (Packages Mall, Al Fatah Mall, Pace Mall, Metro Cash & Carry Store, Anarkali Market, Old Food Street, New Food Street, Cafeterias in Lahore Fort, Cafeterias of the University of Education, Cafeterias of Punjab University, and Cafeterias of the University of Veterinary and Animal Sciences). The study was explained to each participant while collecting data. Permission to collect data was obtained from the management of malls and cafeterias, while in some other open bazaars (markets) like Anarkali Market, Old Food Street, and New Food Street, permission was not needed to collect data. Each participant took 20-25 minutes on average to fill out the questionnaire.

A sample size of 385 participants was calculated using the Kish Leslie formula, considering 5% bond-on error, a 95% confidence interval, and 50% prevalence (for unknown prevalence). To ensure accuracy, the sample size was increased to 500 to account for any missing data or non-response rate.

The questionnaire consisted of demographic characteristics, sources of nutrition information, and knowledge related to nutrition for the target population. The final version of the questionnaire was amended, taking into consideration cultural and religious practices, such as the addition of a question related to alcohol consumption. The main parts of the questionnaire are explained below.

Demographics

The first part of the questionnaire included the demographics of the participants (e.g., age, gender, ethnicity, marital status, occupation, and education level).

Nutrition information

The second part included sources [15] of nutrition information that were divided into four segments: family members, friends and peers, healthcare professionals (e.g., nutritionists, dieticians, doctors, etc.), and online resources (e.g., Google searches, YouTube). [16] Traditional media (e.g., radio, television, newspapers, etc.) and the academic curriculum (schools, colleges, and universities) made the questionnaire easy to fill as the answers were listed on a Likert scale with the options Never, Rarely, Sometimes, and Always. These options were enlisted to assess the sources of nutrition knowledge. The second portion also included the reliability of the nutrition knowledge from the above sources, involving another Likert scale with options of accurate, very reliable, fairly reliable, and unreliable. So, in this portion, the basic purpose of the study was to check where the population is getting nutrition-related knowledge [17] and what their point of view is about the reliability of these sources.

Nutrition knowledge

A total of 15 closed-ended questions were added in the last portion of the questionnaire based on basic nutrition knowledge. Basic nutrition knowledge includes a fact panel, knowledge on appropriate dietary intake, and lifestyle habits [18-19]. There were only two options (zero and one, wherein zero was the wrong answer and one was the right answer), so the range of the lowest and highest possible score by a single participant was designated from zero to 15 [20].

The data at the confidence interval of 95% was analyzed using the IBM Corp. Released 2015. IBM SPSS Statistics for Windows, Version 23.0. Armonk, NY: IBM Corp. [21]. Descriptive analysis was used to analyze the demographic data and for finding the source of nutrition information. The raw score for nutrition knowledge of each individual was calculated to find a mean with the aim of creating a categorical variable; scores above the mean were categorized as high (above 7.5), and scores below the mean were categorized as low (below 7.5). The sources were categorized as 0 for yes and 1 for no in the case of categorical variables.

A bivariate analysis of the nutrition information sources, and knowledge was created by Pearson chi-square; this was with the aim of categorizing the high and low nutrition knowledge values. A logistic regression model was used to find the association between the sources of nutrition information and nutrition knowledge.

## Results

Study sample characteristics

Five hundred people were given the questionnaires, out of which 476 filled out and returned the questionnaires, thus achieving a response rate of 95%. Out of the total 476 participants, 181 (38%) were males and 295 (62%) were females. The mean age of participants was 27.71, wherein 295 (62.22%) of them were non-earning, as most of them were students and some were housewives. All the respondents were Pakistanis, and the majority of them belonged to Lahore. In terms of educational status, most of them (65%) had an education above the 10th grade (matriculation according to the Pakistani education system). Out of all participants, 316 (66.44%) reported being single, and 160 (30.91%) reported being married. The demographic characteristics of participants are given in Table 1 [22].

**Table 1:** Demographic characteristics of participants

Variables	Mean/Percentage (%)
Age	27.7 (mean)
Gender	
Female	62%
Male	38%
Ethnicity	
Punjabi	82.6%
Pathan	7.8%
Sindhi	0.4%
Balochi	0.6%
Others	8.6%
Occupation	
Nonearning	62.2%
Earning	37.8%
Marital status	
Single	66.4%
Married	30.9%
Divorced	1.5 %
Widow	1.3 %
Education	
Matric	14.7 %
Inter	33.4 %
Grad	32.8 %
Others	19.1 %

Sources of nutrition knowledge

**Figure 1:**
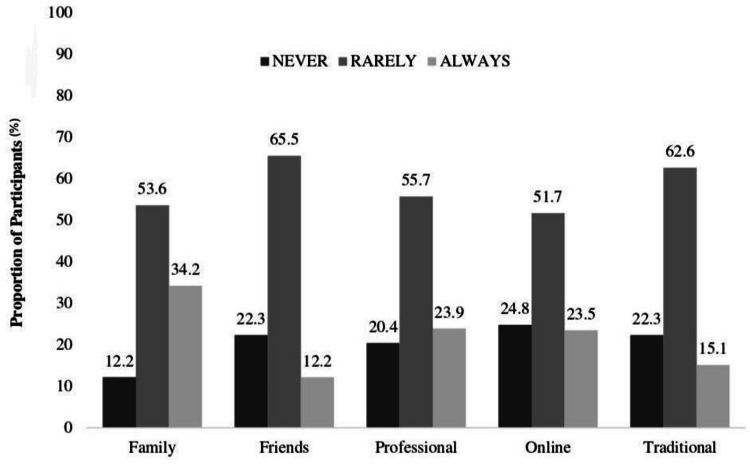
Sources of nutrition knowledge among people in Lahore

Figure 1 explains the different sources of nutrition knowledge that were used by our target population. There were 163 (34.23%) participants who reported taking nutrition-related information from their families, and health professionals were at second place at 113 (23.91%). About 112 (23.51%) participants indicated online sources as their source of information. In addition, 97 (20.41%) and 72 (15.12%) participants chose academic and traditional sources, respectively, and the friend source was at last 58 (12.20%).

Reliability of source of nutrition

**Figure 2:**
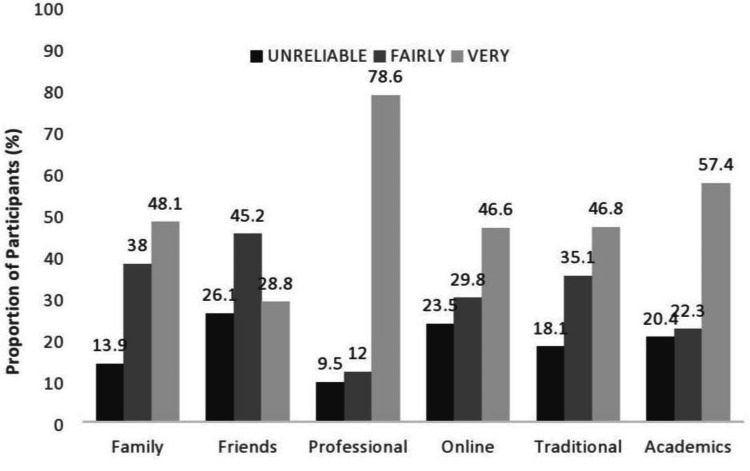
Reliability of sources of nutrition among people in Lahore

Along with the sources of nutrition information, their reliability was also checked through a similar graph. Although most of the people were not taking nutrition information from health professionals, the most reliable source, according to this study, were health professionals (374, or 78.62%). The second most reliable source was academics [273 (57.41%)], while for 229 (48.12%) participants, the reliability source was that of family members. Similarly, for 223 (46.82%) participants, reliable sources were traditional sources, and online sources were the least reliable, as 137 (28.81%) used them (Figure 2).

Basic nutrition knowledge

**Figure 3:**
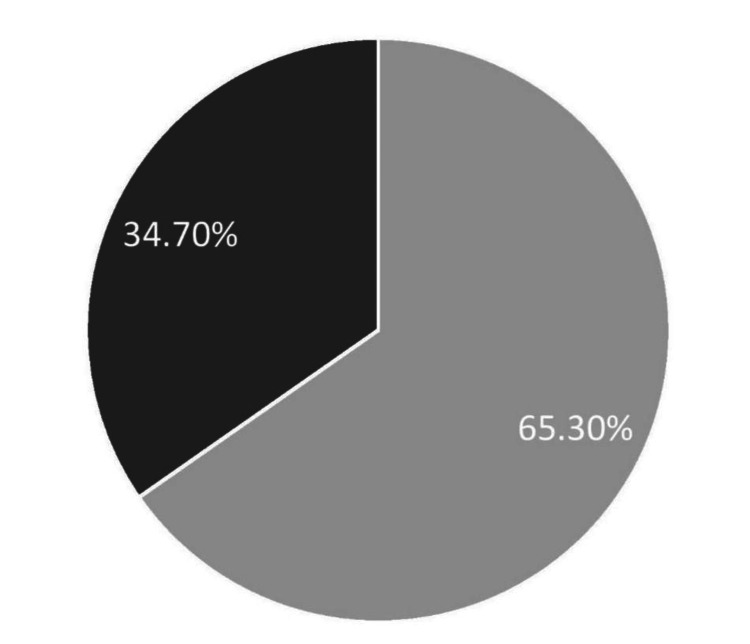
Basic nutrition knowledge among people in Lahore 34.70%: A small amount of nutrition knowledge, 65.30% of the large amount of nutrition knowledge

Figure 3 represents the basic nutrition knowledge of the participants. Out of a total of 15 points, the mean score of 476 participants was 10.89. 

The participants with a large amount of nutrition knowledge were 311 (65.30%), while 165 (34.70%) participants had a small amount of nutrition knowledge (Table 3).

**Table 2:** Basic nutrition knowledge

Source of Nutrition Information	Basic Nutrition Knowledge Low Score	Basic Nutrition Knowledge High Score	P-Value
Family Members			0.027
Yes	137 (83.0%)	281 (90.4%)	
No	28 (175%)	30 (9.6%)	
Friends			0.000
Yes	107 (64.8%)	263 (84.6%)	
No	58 (35.2%)	48 (15.4%)	
Healthcare Professionals			1.000
Yes	131 (79.4%)	248 (79.7%)	
No	34 (20.6%)	63 (20.3%)	
Online Resources			0.000
Yes	98 (59.4%)	260 (83.6%)	
No	67 (40.6%)	51 (16.4%)	
Traditional Media			0.000
Yes	110 (66.7%)	260 (83.6%)	
No	55 (33.3%)	51 (16.4%)	

Although more than half of the participants in this study had a large amount of nutrition knowledge, 287 (60.29%) out of them did not know what nutrition fact panels are, and 271 (56.92%) had no idea what is written on food packages, and they had not tried to read those fact panels [23].

When their means were compared, participants who consulted their friends for nutrition information (9.7 vs. 11.2, p = 0.00) and participants who consulted online sources for nutrition information (9.3 vs. 11.4, p = 0.00) secured more marks in the nutrition knowledge assessment as compared to those who did not use these sources of information. The p-value for these sources was less than 0.05, so there was a significant difference between the results of the participants who were using these sources of nutrition information and those who were not using these sources [24].

After adjusting for age, education, and gender, participants who use online resources had fewer chances (adjusted OR = 0.457; 95% CI: 0.275, 0.759) to acquire low marks in the nutrition knowledge assessment as compared to those who do not use online sources (Table 2) [25].

**Table 3:** Sources of nutrition knowledge

Source	P-value	OR	95% CI
Family	0.238	0.686	0.367, 1.283
Friends	0.016	0.538	0.325, 0.891
Professionals	0.069	1.666	0.962, 2.884
Online	0.002	0.457	0.275, 0.759
Academics	0.006	0.498	0.302, 0.821

## Discussion

This study was aimed at identifying the sources and basic nutrition knowledge among the citizens of Lahore. A multitude of nutritional information sources are found across the globe, with varying reliability. According to 34.2% of participants in our study, family was considered to be the most popular source of nutritional information. As parents are generally the primary source of knowledge throughout life, it is possible that young adults who seek only their family for information and counseling may not yet have developed sufficient health-related skills and inclination to seek the more prominent number of sources. Whereas an Iranian study found the most common resource for seeking health-related information was television and discussion with others [26]. Secondary to family, health care professionals (23.9%) and the internet (23.5%) were also found to be popular sources for seeking health-related information. Digital platforms provide potential for the dissemination of information to many people. For medical issues, 35% of adults in the United States consult the internet to find information and solutions.

This study was carried out mostly in metropolises where the internet is quite accessible; however, in developing countries, internet use is mainly constricted by poor electrical supply, high internet costs, and poor accessibility in remote areas.

Moving towards the reliability of health-related information, healthcare professionals are the most trusted and reliable source of nutrition information. Nonetheless, in developing countries, there is a tradition of self-care and self-medication related to any medical condition. This often results in incorrect diagnoses and potentially adverse effects on health. A previously performed study shows that healthcare professionals are thought to be the most credible source of nutritional information, but many people are exploiting them in favor of browsing the Internet [27]. On the other hand, poor socioeconomic conditions, especially in developing and underdeveloped countries, could also be a reason for not opting for professional help.

Moreover, some of the uneducated people (19%) in our study were incapable of reading the questionnaire. We explained the questionnaire in their mother tongue to uneducated people. Even though nutrition knowledge was higher than average among more than half of the participants, most of them had little to no information about nutrition facts on food packages. Ultimately, it would limit and hinder consumers ability to make an accurate decision regarding the product they are about to choose. In this study, most of the members concurred with the fortification of food items. However, the utilization of dietary supplements among the participants to ensure their wellbeing was less common. On the other hand, a study conducted about attitude, beliefs, information, and application regarding dietary supplements in Saudi Arabia found that more than half of the participants used dietary supplements and were well informed about them [28]. Health promotional activities in the form of commercials, campaigns, and instructive educational seminars can further increase the demands of food fortification. In fact, in a study conducted after participants learned that the principal objective of fortifying vitamin D in milk is to prevent bone-related diseases, about 75% of Mongolians and 18% of Harbin citizens favored obligatory fortification; however, 42% of Harbins agreed upon optional fortification [29].

The research helps establish plausible associations but not a causal relationship between different factors. There was limited time available to conduct the research and collect the data, pertaining to only six months. Therefore, some limitations arose, resulting in the researchers’ inability to comprehend their behavior, knowledge, and level of seriousness regarding nutrition accurately. In addition, there was no data retrieval on the timing or quality of the questionnaire test.

## Conclusions

The present study aimed at assessing nutrition knowledge and identifying the sources of nutrition knowledge among people in Lahore, Pakistan. Most of the participants were not aware of nutritional labels and fact panels. It was also found that those participants who were referred to online resources for information about nutrition knowledge had better results in our questionnaire analysis. There is a need for more extensive research to identify and analyze the quality of the sources that provide nutrition information so that there are better policies and plans integrated and adopted at the community level as well as the national level to increase the overall nutrition knowledge of people.

In addition, the food environment also plays an important role in determining the food choices of people. Although several studies have investigated the impact of knowledge on food choice and consumption habits, they show that subjective knowledge is a stronger driver of consumer behavior than objective knowledge. As one of the factors influencing food choices, nutrition knowledge positively impacts the adoption of healthy eating habits.
